# Case report: Achieving significant tumor reduction in advanced pancreatic adenocarcinoma

**DOI:** 10.3389/fonc.2024.1458517

**Published:** 2024-12-17

**Authors:** Hongying Liu, Yun Wang, Qian Zhang, Nengwen Ke

**Affiliations:** ^1^ Department of Pancreatic Surgery, West China Hospital, Sichuan University/West China School of Nursing, Sichuan University, Chengdu, China; ^2^ Chengdu Shang Jin Nan Fu Hospital, Shang Jin Hospital of West China Hospital, Sichuan University, Chengdu, China; ^3^ Department of Gastrointestinal surgery, The Second People’s Hospital of Qujing City, Qujing, China; ^4^ Department of Pancreatic Surgery, West China Hospital, Sichuan University, Chengdu, China

**Keywords:** pancreatic adenocarcinoma, tumor reduction, case report, immunotherapy, chemotherapy

## Abstract

Pancreatic cancer remains a highly malignant and challenging tumor with a dismal 5-year survival rate of only 13%. The majority of patients are diagnosed at advanced stages, where surgical options are limited, and prognosis is poor. Immunotherapy, particularly PD-1 inhibitors, has shown limited success in pancreatic cancer due to its unique tumor immune microenvironment. However, certain genetic profiles, such as BRCA1/2 mutations, high tumor mutational burden (TMB), or microsatellite instability-high (MSI-H), may enhance sensitivity to these therapies. This report presents two cases of advanced pancreatic cancer with BRCA1/2 mutations treated with a combination of chemotherapy and immune checkpoint inhibitors. The first patient, with TMB-H and stable microsatellites, achieved complete remission after conversion therapy and remains disease-free for over two years post-surgery. The second patient, with MSI-H and low TMB, experienced significant tumor regression and improved quality of life with a prolonged progression-free survival, although the patient ultimately declined surgery. These cases suggest that combined chemotherapy and immunotherapy may offer a promising treatment option for select pancreatic cancer patients, particularly those with specific genetic profiles, warranting further investigation into personalized approaches to immunotherapy in this malignancy.

## Introduction

Pancreatic cancer is a highly malignant tumor of the digestive system with a 5-year survival rate of only 13% (1). It often lacks specific symptoms in the early stages, grows rapidly, and is prone to metastasis and recurrence. Most patients are diagnosed at an advanced stage with low surgical resection rates and poor prognosis. Previously, the special tumor immune microenvironment of pancreatic cancer was thought to result in low drug sensitivity (2). As a result, PD-1 and other immune checkpoint inhibitors have not been broadly successful in treating pancreatic cancer (3, 4). However, emerging evidence suggests that certain genetic profiles, such as BRCA1/2 mutations, high tumor mutational burden (TMB-H), or microsatellite instability-high (MSI-H), may confer increased sensitivity to these therapies. This report presents two cases of advanced pancreatic cancer with BRCA1/2 mutations, who were treated with a combination of chemotherapy and immune checkpoint inhibitors. The outcomes suggest that such combined therapy may offer a viable treatment option for select patients with advanced disease. This manuscript follows the CARE guidelines for case reports to ensure the accuracy and transparency of the presented data.

## Case presentation

### Case 1

A 33-year-old male presented with a two-month history of abdominal pain, which had worsened over the past 20 days, accompanied by jaundice and melena. He had no significant past medical history and no relevant family history. Enhanced abdominal computed tomography (CT) revealed a slightly hypodense mass in the right lobe of the liver, measuring approximately 6.9 cm x 6.1 cm, with blurred edges, faint lobular changes, heterogeneous density, and marked ring enhancement during the arterial phase. Additionally, a homogeneously hypodense soft tissue mass was identified in the head of the pancreas, measuring about 5.4 cm x 3.4 cm, with ring enhancement (**Supplementary Figure S1A**). This pancreatic mass raised the suspicion of metastasis or primary pancreatic cancer. Laboratory tests showed the following results: alpha-fetoprotein (AFP) at 8.48 ng/ml, carcinoembryonic antigen (CEA) at 176 ng/ml, and serum carbohydrate antigen 19-9 (CA 19-9) exceeding 1000 U/ml. Based on imaging and tumor marker findings, a preliminary diagnosis of a malignant tumor in the pancreatic head with liver metastasis was made. The TNM staging was determined to be T2N1M1, Stage IV. Given the advanced nature of the pancreatic cancer, the patient was not a candidate for surgical intervention.

**Figure 1:**
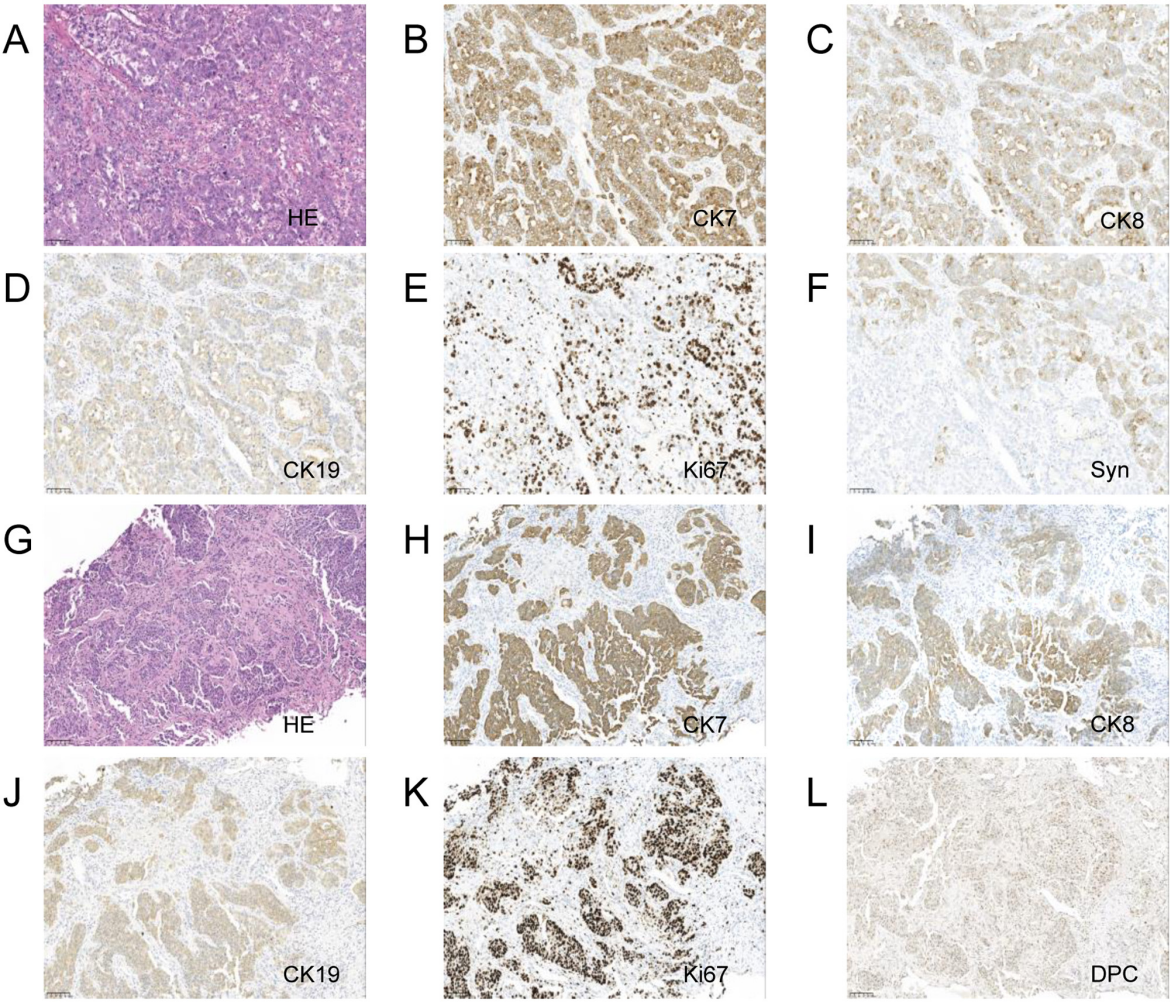
Pancreas and liver biopsy 20x light microscope image of case 1. **(A–F)** respectively shows 20x light microscope images of pancreas biopsy tissues HE, CK7, CK8, CK19, Ki67, Syn. **(G–L)** respectively shows 20x light microscope images of liver biopsy tissues HE, CK7, CK8, CK19, Ki67, DPC.

A laparoscopic biopsy of the liver and pancreas was performed. Postoperative pathology revealed poorly differentiated adenocarcinoma. Immunohistochemical staining of the liver biopsy was positive for CK7, CK8/18, CK19, and negative for Syn, CgA, P63, CK5/6, Heppar-1, GPC3, GS (+), TTF-1, CDX-2, SATB-2, DPC4 (±), Ki-67 (+, 80%), supporting the diagnosis of adenocarcinoma (**Figure 1**). Given the clinical presentation, the findings were consistent with liver metastasis from pancreatic cancer. Genetic analysis revealed the following: Microsatellite Stable (MSS) status and a Tumor Mutational Burden (TMB) of 9.84 mutations/Mb (high). Somatic mutations were identified in genes including TARX, BRCA1, BRCA2, BRIP1, CHD1, EPHA7, ERCC3, ETV1, FANCD2, INPP48, LRP18, MSH3, PIK3C2G, PTEN, RASA1, SMAD2, STAG2, STAT58, TAP2, and WRN.

The patient then received three cycles of neoadjuvant chemotherapy combined with immunotherapy over three months: Gemcitabine 1000 mg/m^2^ on days 1 and 8, Cisplatin 25 mg/m^2^ on days 1 and 8, and Camrelizumab 200 mg on day 1, every three weeks (Q3W). Following this treatment regimen, an enhanced abdominal CT scan revealed a slightly hypodense nodule in the right lobe of the liver, measuring approximately 2.3 x 2.8 cm with inhomogeneous density and unclear boundaries, displaying uneven enhancement (**Supplementary Figure S1B**). Additionally, a hypoechoic mass in the head of the pancreas, measuring about 2.0 x 2.2 cm, showed significant reduction in size compared to previous scans. Laboratory tests showed AFP at 9.15 ng/ml, CEA at 6.41 ng/ml, and CA 19-9 at 90.10 U/ml. The primary tumor and metastatic lesions had reduced by more than 30%, and the therapeutic effect was assessed as Partial Response (PR) according to the Response Evaluation Criteria in Solid Tumors (RECIST). Given the patient’s young age and significant response to conversion therapy, he was readmitted for surgical treatment.

**Figure 2:**
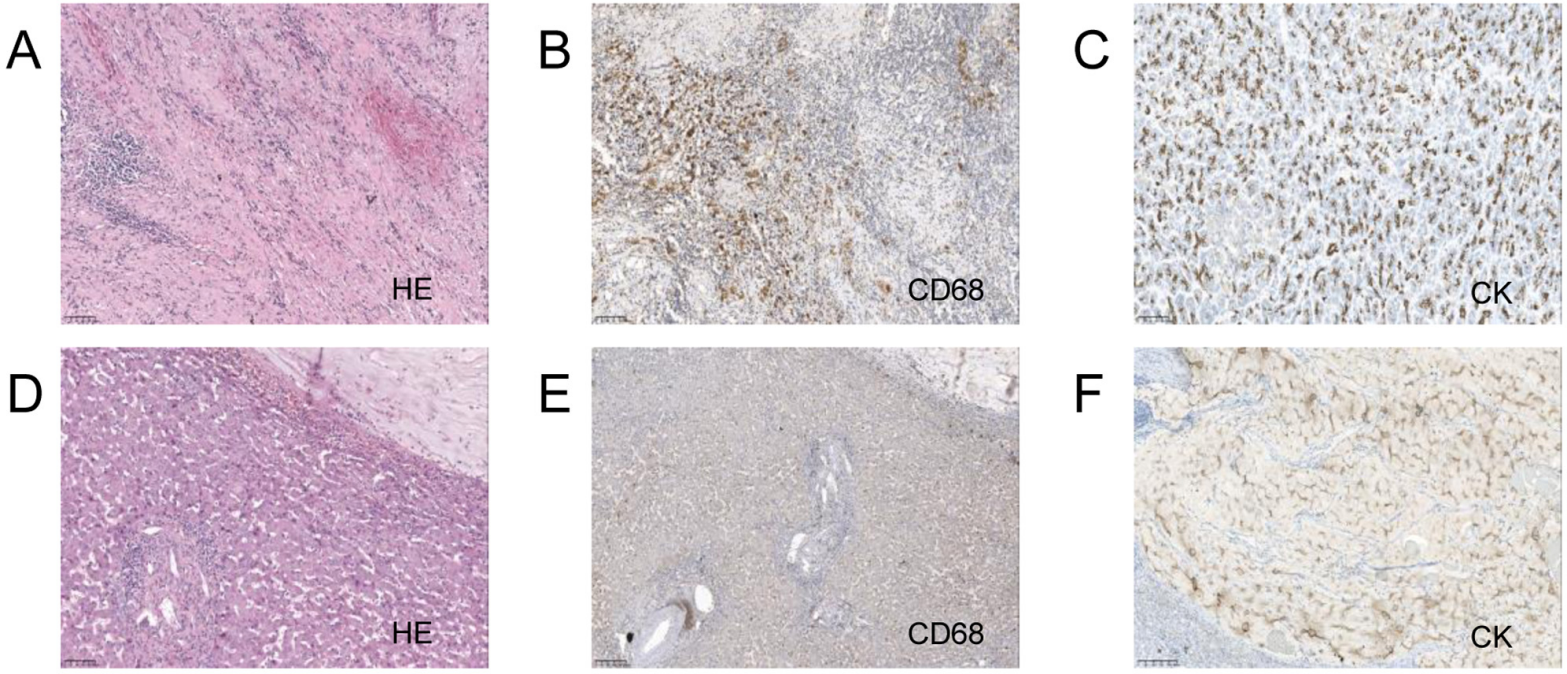
Pancreaticoduodenectomy and right hepatectomy tissue 20x light microscope image of case 1. **(A–C)** respectively shows 20x light microscope images of pancreas tissues HE, CD68, CK. **(D–F)** respectively shows 20x light microscope images of liver tissues HE, CD68, CK.

After multidisciplinary discussion and consultation, the patient underwent a pancreaticoduodenectomy and right hepatectomy under general anesthesia. Postoperative pathology indicated fibrous tissue proliferation, hyaline degeneration, and foam cell reaction in the “gray-white area” of the pancreaticoduodenectomy specimen, consistent with post-treatment appearance, with no definitive cancer residue, and surrounding tissue showing chronic inflammatory changes (**Figures 2A, B**). The liver specimen showed numerous mucinous lakes with no definitive cancer components, also consistent with post-treatment appearance (**Figures 2C, D**). **Table 1** shows the lab results for pre and post treatment. The patient was followed up for two years, and no metastasis or recurrence was observed during the latest review (**Supplementary Figure S1C**). **Supplementary Figure S2** shows the treatment timeline of this patient.

**Table 1:** Lab results for pre and post treatment.

	Case 1		Case 2	
	Pre	Post	Pre	Post
ALT IU/L	23	74	123	64
AST IU/L	22	47	49	38
CA125 U/ml	1451	14.9	13.2	8.03
CA199 U/ml	>1000	53	19.2	10.5
CEA ng/ml	200	3.95	2.73	2.96
AFP ng/ml	7.46	9.19	6.12	6.36
TB umol/L	56.6	22.1	42.6	10.5
HB g/L	74	111	158	168
WBC 10^9^/L	7.4	1.94	8.63	8.81
PLT 10^9^/L	179	70	220	198

ALT, Alanine aminotransferase; AST, Aspartate transaminase; CA125, Carbohydrate antigen 125; CA199, Carbohydrate antigen 199; CEA, Carcinoembryonic antigen; AFP, Alpha-fetoprotein; TB, Total bilirubin; HB, Hemoglobin; WBC, White blood cell; PLT, Blood platelet.

### Case 2

A 48-year-old male was admitted to our department with abdominal distension, accompanied by skin and scleral icterus for over a month, and had undergone percutaneous transhepatic biliary drainage for two weeks. An enhanced abdominal CT scan revealed a slightly low-density mass in the pancreatic head, with blurred edges and a maximum cross-sectional area of approximately 4.2 x 3.4 cm. The enhancement was less than that of the pancreatic parenchyma, and the mass encircled the superior mesenteric vessels, causing significant stenosis of the superior mesenteric vein, slight thickening of the lower branches, and a rough edge of the superior mesenteric artery. The lesion was indistinct from the duodenum, with mild dilation of the main pancreatic duct, suggesting the possibility of pancreatic cancer with suspected invasion of the superior mesenteric vein (**Supplementary Figure S3A**). He had no significant past medical history and no relevant family history.

**Figure 3:**
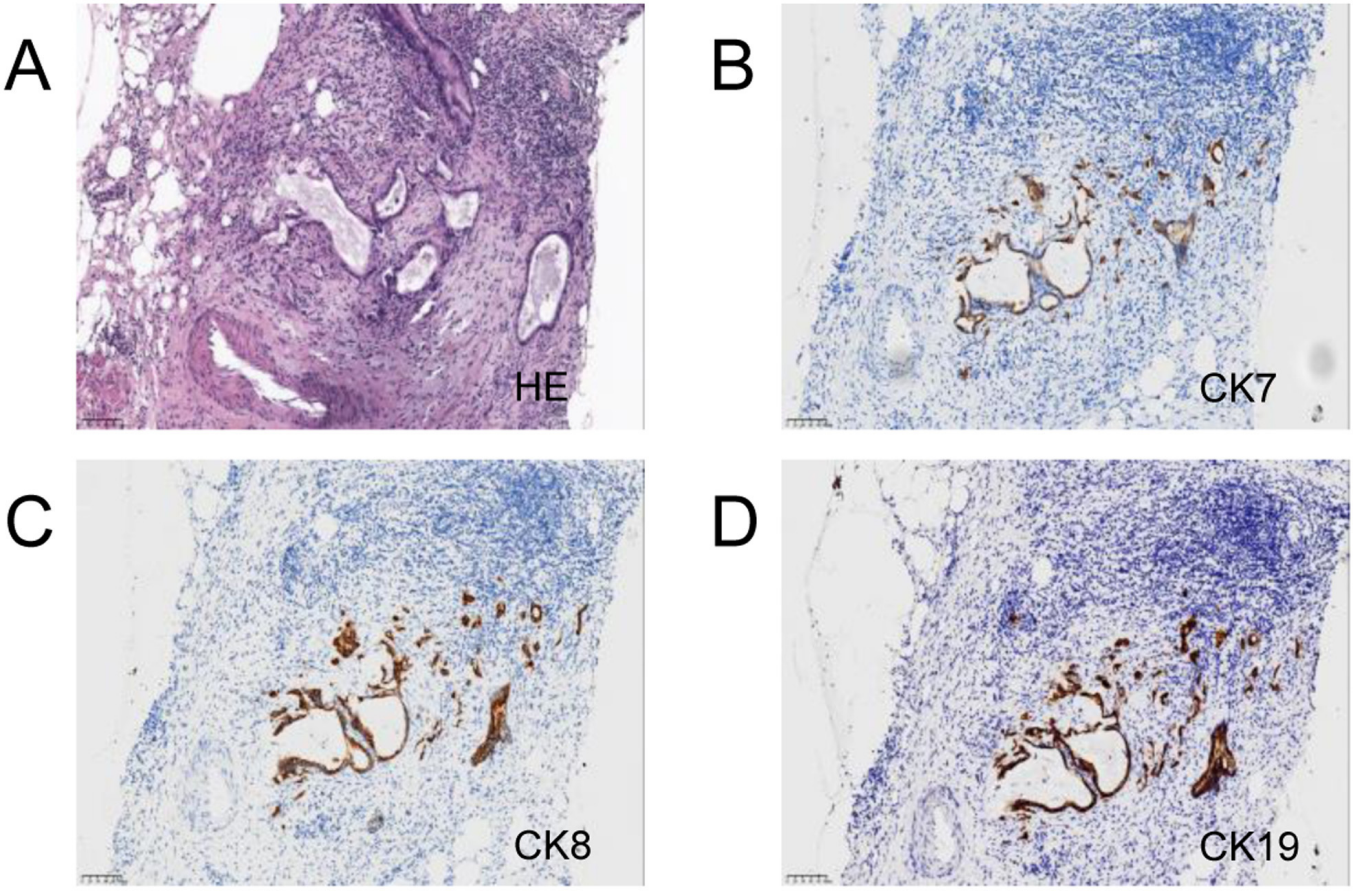
Pancreas and liver biopsy 20x light microscope image of case 2. **(A–C)** respectively shows 20x light images of pancreas tissues HE, CD68, CK. **(D–F)** respectively shows 20x light images of liver tissues HE, CD68, CK.

After multidisciplinary discussion, curative surgery was deemed unfeasible. A laparoscopic biopsy of the pancreatic mass was performed, and intraoperative findings included no nodular changes on the liver surface, a small amount of abdominal effusion, and a hard mass protruding from the head of the pancreas. The biopsy of the mass indicated atypical glands, suggesting adenocarcinoma (**Figure 3**). Genetic testing for immune-related markers showed MSI-H and a tumor mutational burden (TMB) of 3.84 (low). Somatic mutations were identified in genes including BRCA1, BRCA2, BRIP1, CHD1, EPHA7, ERCC3, ETV1, FANCD2, INPP48, LRP18, MSH3, PIK3C2G, PTEN, RASA1, SMAD2, STAT58, and TAP2.

The patient underwent his first FOLFIRINOX chemotherapy session on June 24, 2021, followed by a total of 10 sessions, but discontinued chemotherapy due to side effects, with the last session on October 22, 2022.The specific medication regimen (based on a body surface area of 1.90 m²) was as follows: oxaliplatin 80 mg/m^2^, irinotecan 170 mg/m^2^, calcium folinate 315 mg/m^2^, and fluorouracil 400 mg/m^2^ administered on day 1 via intravenous infusion, followed by fluorouracil 4250 mg administered continuously by micro-pump over 46 hours. During the same period, the patient received immunotherapy with Camrelizumab 200 mg every three weeks (q3w). Due to the patient’s inability to tolerate the side effects of chemotherapy and reluctance to continue, they received 14 cycles of immunotherapy alone from January 20, 2022, to October 22, 2022.

Enhanced CT scans were conducted every three months during the treatment period, showing continuous tumor regression. Initially, the tumor encircled the superior mesenteric artery (SMA) by more than 270 degrees (**Supplementary Figure S3A**). By June 2023, enhanced abdominal CT scans indicated that the tumor’s contact with the SMA had reduced to less than 90 degrees (**Supplementary Figures S3B-D**). The patient’s physical condition improved significantly, with complete resolution of jaundice, disappearance of abdominal pain, and weight gain. Surgical treatment was recommended, but the patient refused. The patient declined surgery, but the disease progressed, leading to four additional cycles of immunotherapy with Camrelizumab 200 mg every three weeks (Q3W) from October 13, 2023, to December 15, 2023. The patient underwent PTCD for jaundice on November 10, 2023. On December 29, 2023, under general anesthesia, the patient underwent laparoscopic gastrojejunostomy, Roux-en-Y hepaticojejunostomy, cholecystectomy, and adhesiolysis. From April 25, 2024, to August 15, 2024, the patient received five cycles of Atezolizumab 1200 mg every three weeks (Q3W). **Supplementary Figure S4** shows the treatment timeline of this patient.

## Discussion

Pancreatic cancer is an aggressive and prevalent malignancy of the digestive tract, characterized by the highest mortality and lowest survival rates among all digestive tract cancers, with a 5-year survival rate of only 13% (1). Due to its deep anatomical location and the absence of early diagnostic methods, 70% to 80% of pancreatic cancer patients are diagnosed at a locally advanced or metastatic stage, with only 10% suitable for surgical resection (5, 6). Previous studies have shown that pancreatic cancer patients with liver metastasis generally have a total survival period not exceeding six months, even with active treatment (7).

Following treatment with chemotherapy and immunotherapy, the Case 1 patient have been proved complete response by postoperative pathology, and the patient has remained disease-free for over 24 months. We attribute the favorable treatment outcome in this case to the patient’s genetic profile, which revealed multiple gene mutations, particularly BRCA mutations. Studies have demonstrated that patients with BRCA mutations are sensitive to platinum-based chemotherapy (8), leading us to employ a chemotherapy regimen of gemcitabine and cisplatin. Furthermore, the cut off value of high TMB are varies among studies. It is 10 mutations/Mb in Quintanilha’s study (9). However, 5 mutations/Mb is the cut off value of high TMB in Hatakeyama (10) and Imamura studies (11). Therefore, we describe this case with a high TMB (9.84 mutations/Mb). Due to previous study confirmed that high TMB is associated with sensitivity to immunotherapy (9), we incorporated a PD-1 immune checkpoint inhibitor into the treatment.

In another advanced case with BRCA mutations, the patient exhibited low TMB and MSI-H, an indicator of sensitivity to immunotherapy (12). Consequently, we administered a treatment regimen of FOLFIRINOX plus PD-1, which resulted in significant tumor regression, resolution of jaundice, and substantial improvement in quality of life. Although the patient ultimately refused surgery and the disease progressed, a progression-free survival of 36 months was achieved. While MSI-H is often associated with a high TMB due to the accumulation of mutations from mismatch repair deficiencies. Some MSI-H tumors may exhibit a low TMB, potentially due to specific genetic mutations or unique tumor biology. MSH3, a component of the mismatch repair (MMR) system, a major source for the inactivation in MSI frameshift events (13). Mutations in MSH3 can lead to MSI-H. However, the impact on TMB might be more nuanced. It is possible that MSH3 mutations result in selective repair deficiencies, affecting only certain types of DNA errors while not significantly increasing the overall mutation burden. Additionally, other compensatory repair mechanisms may mitigate the expected increase in TMB despite the presence of MSI-H.

Previous clinical studies have shown that the overall efficacy of immunotherapy in treating pancreatic cancer is limited. For example, the overall response rate of PD-1 antibody monotherapy or combined CTLA-4 antibody therapy is only 0.10%-12% and 3%, respectively (3, 4, 11–15). Despite numerous clinical trials on immune checkpoint inhibitors, monoclonal antibodies against key antigens, and immune cell therapies, satisfactory clinical benefits have not been realized. The immune microenvironment of pancreatic cancer is highly heterogeneous, posing significant challenges to immunotherapy (2). However, some patients can still benefit from immunotherapy, particularly those with MSI-H or high TMB. A study by the University and Hospital Trust of Verona analyzed data from 8,323 patients with pancreatic adenocarcinoma, finding that only 1% and 2% of patients had MSI-H and high TMB, respectively (16).

BRCA mutations are associated with defects in the DNA repair pathway, potentially increasing the mutation burden in tumor cells. Therefore, BRCA mutations may have potentially pathogenic in both cases. Some studies indicate that the combination of high TMB with BRCA mutations may lead to immune activation within the tumor microenvironment, characterized by higher levels of T lymphocyte infiltration and increased PD-L1 expression (17). These factors may enhance the efficacy of immunotherapy. Both patients significant responded to the combination of chemotherapy and immunotherapy in present study may as evidence for that.

A recent report from Memorial Sloan Kettering Cancer Center indicates that patients with BRCA2 mutations are more responsive to immune checkpoint blockade (ICB). Interestingly, this benefit is observed primarily in patients with tumors not typically rich in homologous recombination deficiency (HRD), such as melanoma and small cell lung cancer, whereas patients with HRD-related tumors (breast, prostate, pancreatic, or ovarian cancers) did not benefit as much from ICB (18).

Additionally, we know that it is difficult to determine the role of immunotherapy in the clinical outcome of two cases with different MSI and TMB statuses. However, both of them were responded well to chemotherapy combined with immunotherapy. One patient had MSI-H with low TMB, while the other had high TMB with stable microsatellites. This phenomenon aligns with findings that BRCA mutations rarely occur in the background of MSI-H but are more common with high TMB (17). This observation is also related to WRN gene mutations, which are closely associated with high TMB (19). In our reported cases, one patient had a WRN mutation, while the other did not. Despite the different MSI and TMB statuses, both patients achieved favorable outcomes with chemotherapy and immunotherapy. Therefore, we suggest that screening pancreatic cancer patients for MSI-H or high TMB may be an effective strategy for identifying candidates for immunotherapy.

## Data Availability

The raw data supporting the conclusions of this article will be made available by the authors, without undue reservation.
