# Supplementary material for: Case report: Achieving significant tumor reduction in advanced pancreatic adenocarcinoma
Source: Front Oncol. 2024 Dec 17;14:1458517. doi: 10.3389/fonc.2024.1458517 (PMC11685077; doi:10.3389/fonc.2024.1458517)
Supplement: Supplementary file 1 [file DataSheet1.docx]

**Supplement materials**

**Supplement Figure 1 Contrast-enhanced abdominal CT during treatment.**


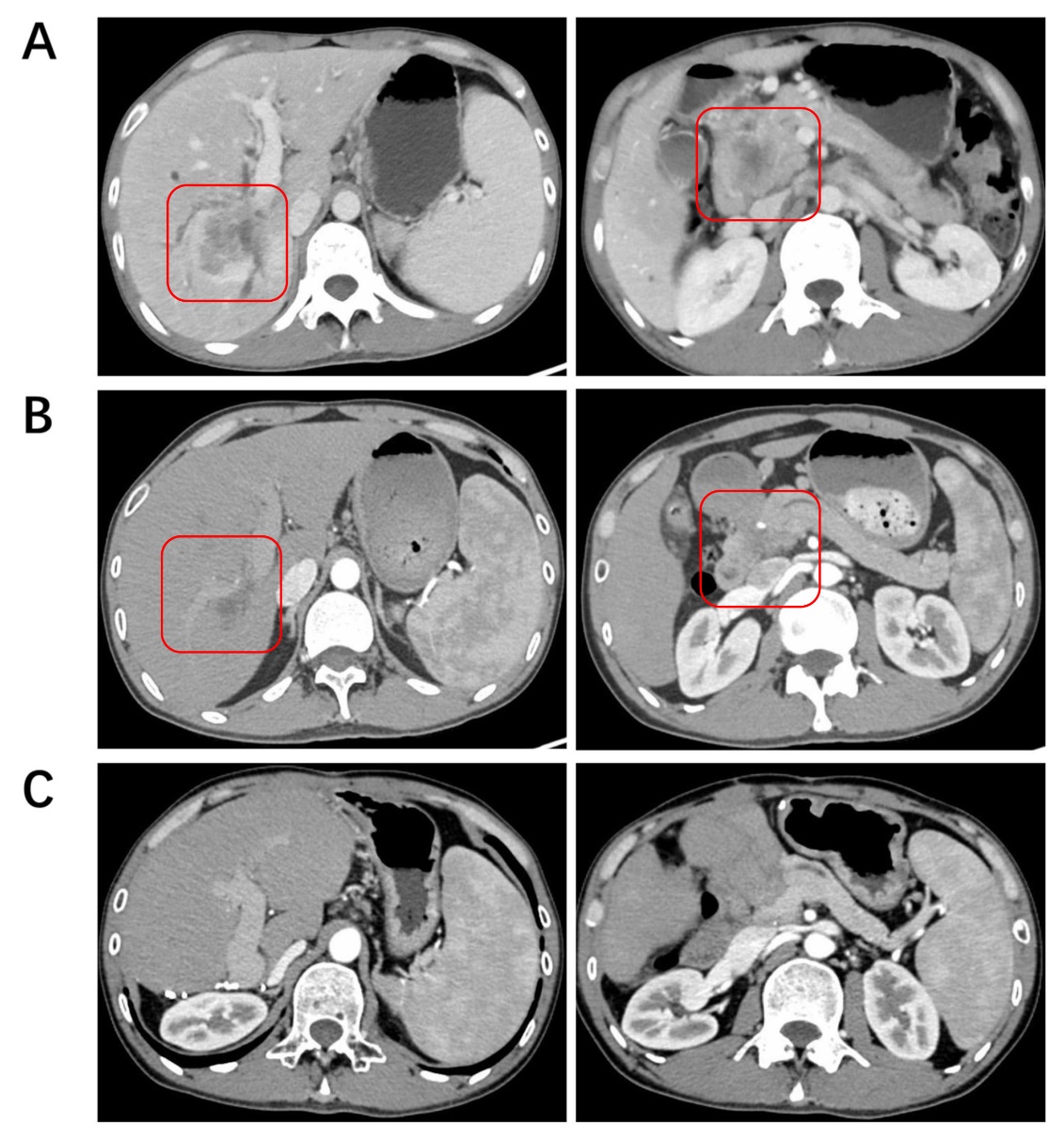


A: Pretreatment. A slightly hypodense mass in the right lobe of the liver, measuring approximately 6.9cm x 6.1cm in cross-section. A homogeneously hypodense soft tissue mass was observed in the head of the pancreas, measuring about 5.4cm x 3.4cm, with ring enhancement. B: 3 months after three courses of neoadjuvant chemotherapy plus immunotherapy over the next four months: GP scheme + Camrelizumab, the matastiss of liver size was significantly reduced and limited to 2.3 x 2.8cm, and the tumor of head pancreas was reduced to 2.0 x 2.2cm. C: 24 months after a pancreaticoduodenectomy and right hepatectomy, and no metastasis or recurrence was seen on current review.

**Supplement figure 2 Treatment timeline of case 1**


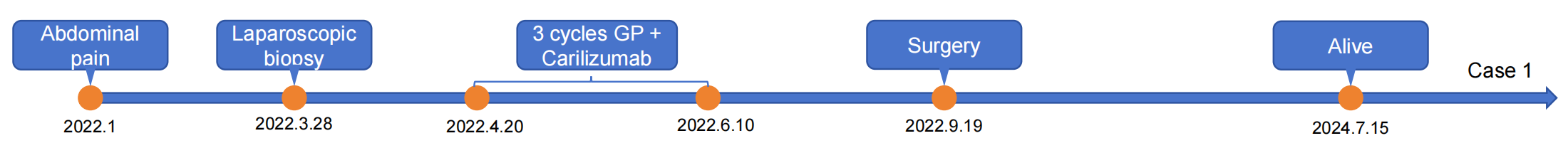


**Supplement Figure 3 Contrast-enhanced abdominal CT during treatment.**


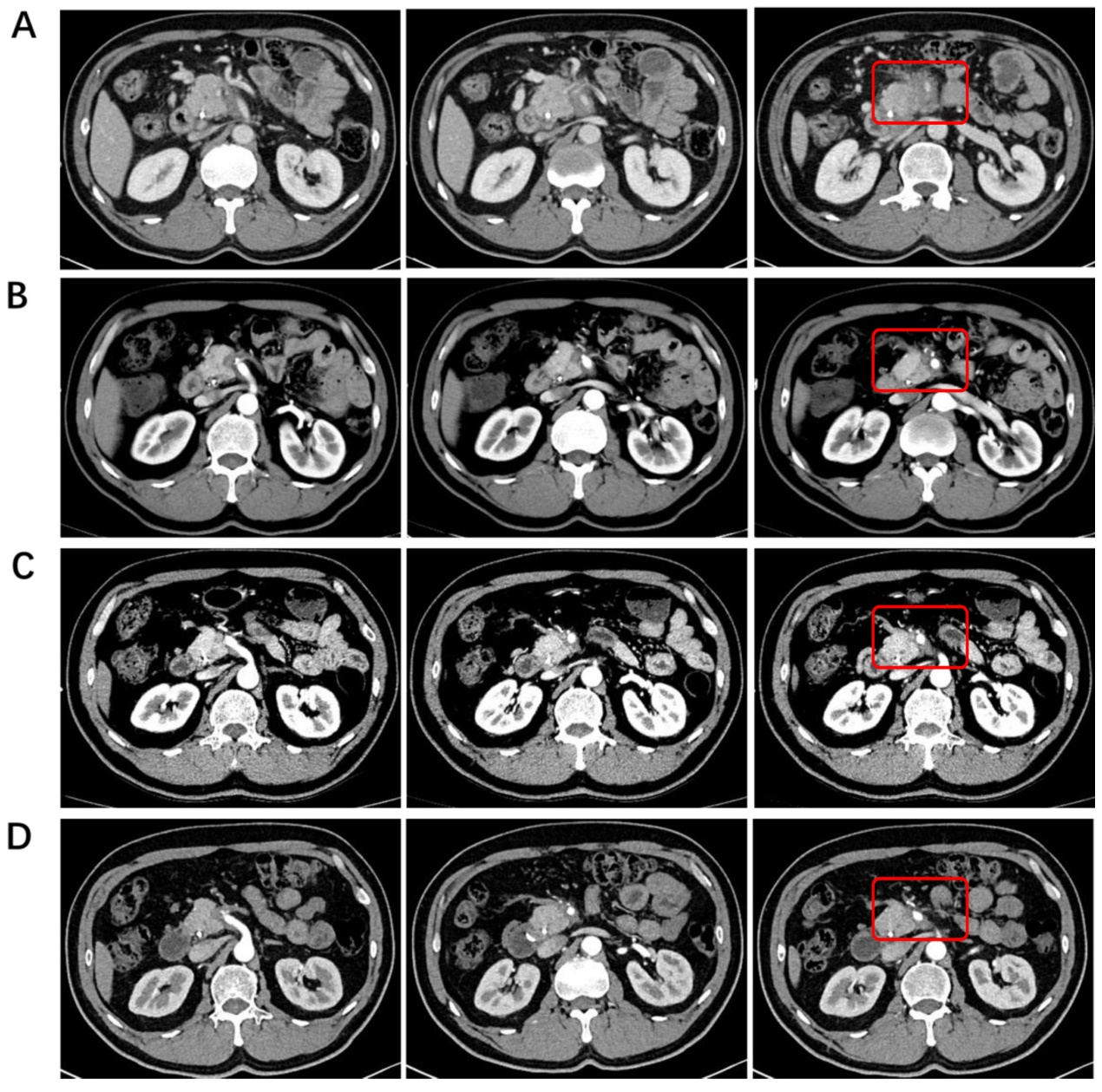


A: Pretreatment. a slightly low-density mass in the pancreatic head, with blurred edges and a larger cross-sectional area of about 4.2 x 3.4 cm. The enhancement was lower than the pancreatic parenchyma, with the superior mesenteric vessels encircled by the mass. B: 6 months after six courses of FOLFIRINOX chemotherapy plus Camrelizumab immunotherapy, the tumor size was significantly reduced and limited to 1.7cmx1.3cm. the tumor was in contact with the superior mesenteric artery for less than 90 degrees. C: 12 months after ten courses of FOLFIRINOX chemotherapy plus Camrelizumab immunotherapy, the tumor size was significantly reduced and limited to 1.3cmx1.1cm. D: 24 months after ten courses of FOLFIRINOX chemotherapy plus Camrelizumab immunotherapy, the tumor size was significantly reduced and limited to 1.3cmx1.1cm.

**Supplement figure 4 Treatment timeline of case 2**


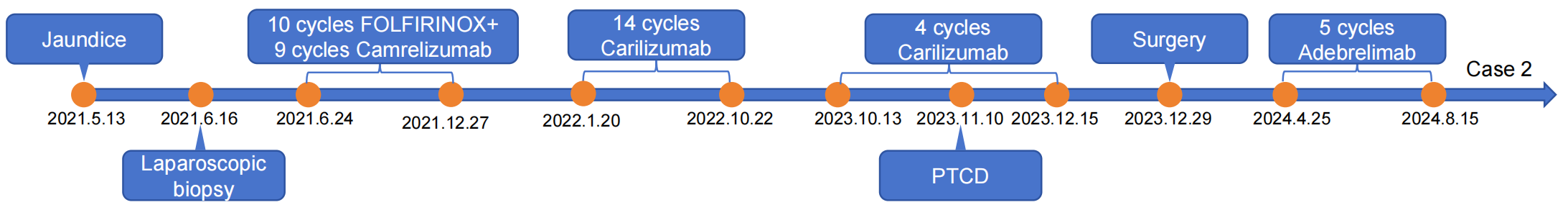


**Detail therapeutic regimens of case 1.**

The patient received chemotherapy combined with immunotherapy on April 20, 2022, May 15, 2022, and June 10, 2022. The specific regimen was as follows: Gemcitabine 1000 mg/m² on Day 1, Cisplatin 25 mg/m² on Days 1 and 8, and Camrelizumab 200 mg on Day 1, administered every three weeks (Q3W).

**Detail therapeutic regimens of case 2.**

The patient underwent 10 cycles of FOLFIRINOX chemotherapy from June 24, 2021, to December 27, 2021. The specific medication regimen, based on a body surface area of 1.90 m², was as follows: Oxaliplatin 80 mg/m², Irinotecan 170 mg/m², Calcium Folinate 315 mg/m², and Fluorouracil 400 mg/m² administered on Day 1 via intravenous infusion, followed by a continuous 46-hour infusion of Fluorouracil 4250 mg via micro-pump. During the same period, the patient also received nine cycles of immunotherapy with Camrelizumab 200 mg every three weeks (Q3W).

Due to the patient's inability to tolerate the side effects of chemotherapy and reluctance to continue, they received 14 cycles of immunotherapy alone from January 20, 2022, to October 22, 2022. The patient declined surgery, but the disease progressed, leading to four additional cycles of immunotherapy with Camrelizumab 200 mg every three weeks (Q3W) from October 13, 2023, to December 15, 2023. The patient underwent PTCD for jaundice on November 10, 2023. On December 29, 2023, under general anesthesia, the patient underwent laparoscopic gastrojejunostomy, Roux-en-Y hepaticojejunostomy, cholecystectomy, and adhesiolysis. From April 25, 2024, to August 15, 2024, the patient received five cycles of Atezolizumab 1200 mg every three weeks (Q3W).
